# Paleoproterozoic high-pressure metamorphism in the northern North China Craton and implications for the Nuna supercontinent

**DOI:** 10.1038/ncomms9344

**Published:** 2015-09-21

**Authors:** Bo Wan, Brian F. Windley, Wenjiao Xiao, Jianyun Feng, Ji'en Zhang

**Affiliations:** 1 State Key Laboratory of Lithospheric Evolution, Institute of Geology and Geophysics, Chinese Academy of Sciences, Beijing 100029, China; 2 CAS Center for Excellence in Tibetan Plateau Earth Sciences, Beijing 100101, China; 3 Department of Geology, University of Leicester, Leicester LE1 7RH, UK; 4 Xinjiang Research Center for Mineral Resources, Xinjiang Institute of Ecology and Geography, Chinese Academy of Sciences, Urumqi 830011, China; 5 Petroleum Exploration and Production Research Institute, SINOPEC, Beijing 100083, China

## Abstract

The connection between the North China Craton (NCC) and contiguous cratons is important for the configuration of the Nuna supercontinent. Here we document a new Paleoproterozoic high-pressure (HP) complex dominated by garnet websterite on the northern margin of the NCC. The peak metamorphism of the garnet websterite was after ∼1.90 Ga when it was subducted to eclogite facies at ∼2.4 GPa, then exhumed back to granulite facies at ∼0.9 GPa before ∼1.82 Ga. The rock associations with their structural relationships and geochemical affinities are comparable to those of supra-subduction zone ophiolites, and supported by subduction-related signatures of gabbros and basalts. We propose that a ∼1.90 Ga oceanic fragment was subducted and exhumed into an accretionary complex along the northern margin of the NCC. Presence of the coeval Sharyzhalgai complex with comparable HP garnet websterites in the southern Siberian active margin favours juxtaposition against the NCC in the Paleoproterozoic.

## Abstract (web summary)

Identifying past continental configurations is important for our understanding of the Earth system. Here, the authors present mapping and geochemical analyses that connect the North China Craton with other continents, with implications for the Nuna/Columbia supercontinent configuration at 1.8 Ga.

## Graphical abstract

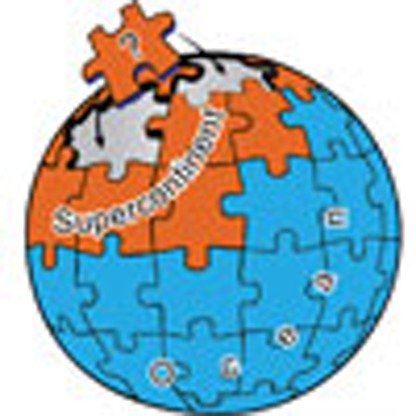

Dispersed continents can merge by plate tectonics into a single supercontinent^1^, a global cyclical assembly that plays a key role in mantle convection, continental growth, ore formation, atmospheric composition and climate-environmental changes^2^,^3^,^4^,^5^,^6^. A key basic method to reconstruct a former supercontinent is to compare geologically similar piercing points or diagnostic belts between cratons. Using such a framework, a proposed close connection between five cratons at ∼1.8 Ga including Baltica-North America-Siberia-Northern and Western Australia was termed ‘Nuna'^7^. Later configurations were consistent with Baltica-North America-Siberia in the core of Nuna (Fig. 1a), but there has been little agreement about the relationships between these three continents and other cratons^8^,^9^,^10^,^11^,^12^. In particular, the North China Craton (NCC) has been variably interpreted to be in direct contact either with Baltica^13^, North America^14^, Siberia^15^, India^10^ or Amazonia^16^.

The NCC is one of the oldest cratons in the world because of the presence of >3.8 Ga rocks^17^, and 2.55–2.50 Ga HP mafic granulites^18^. Although it is important to understand the NCC in the context of ancient supercontinent reconstructions, most studies have focused on the timing and mechanism of amalgamation of the NCC. A recent popular model is illustrated in Fig. 1b: four Neoarchean blocks mutually collided to form the Khondalite, Trans-North China and Jiao-Liao-Ji orogenic belts^19^, where extensive HP metamorphic rocks record a highest pressure of ∼1.6 GPa^20^,^21^,^22^. Other models have mainly focused on the number of blocks, their subduction polarity and the terminal collision time of the Trans-North China orogen^23^,^24^,^25^,^26^. To improve current understanding, a more detailed study of the Paleoproterozoic history of the craton margins is urgent, because they are potential locations that provide constraints on craton-connecting events. However, understanding the Paleoproterozoic histories of the margins of the NCC is hampered by overprinting by Phanerozioc orogenic events (Fig. 1b). A few Paleoproterozoic metamorphic rocks between the Yinshan and Longgang blocks on the northern margin of the NCC (Fig. 1b) are regarded as part of the North-South (NS)-trending Trans-North China orogen^19^,^23^, whereas they may be part of an East-West (EW)-trending orogenic belt^25^. Clearly, the Paleoproterozoic geology in the margins of the craton is controversial, and thus requires a more detailed study to clarify and constrain the connections between the NCC and other cratons.

In this study, we focus on the Alxa complex on the northernmost margin of NCC, where several North-West-South-West (NW-SW) trending websterite-gabbro lenses (±garnet, Grt) occur in Paleoproterozoic gneisses^27^ (Figs 1b and 2a). We report for the first time a new Paleoproterozoic HP complex dominated by Grt-websterite that is neither close to the known HP rocks nor in the same structural zones within the NCC (Fig. 1b). Structural, mineralogical and geochronological evidence indicates that an exhumed Paleoproterozoic accretionary complex containing oceanic fragments occurs along the northern margin of the NCC. These findings place firm constraints on the position of the NCC in the framework of a reconstructed Paleoproterozoic Nuna/Columbia supercontinent.

## Results

### The Alxa complex

The Alxa complex is mostly covered by Quaternary sediments of the Tengger Desert, but the limited outcrops are well-exposed. The complex mostly comprises Paleoproterozoic (and a few Mesoproterozoic) gneisses that contain ultramafic-mafic lenses up to a kilometre long and wide that are broadly aligned parallel to the regional East-North-East-West-South-West (ENE-WSW) trend^27^ (Supplementary Fig. 1). Although the ultramafic-mafic rocks were earlier mapped as ‘intrusions'^27^, we have not observed any intrusive relationships between them and the surrounding conformable gneisses. Most importantly, the presence of considerable metamorphic Grt in the websterites is potentially indicative of HP metamorphism. Figure 2a–c illustrate their field relationships, and below we present the petrology of the Grt-websterites and adjacent rocks.

Widespread migmatitic granitic gneisses are characterized by compositional bands (Fig. 3a) of plagioclase (Pl)+quartz (Qtz)+biotite (Bt), and amphibole (Amp)+pyroxene+Grt. The granitic gneisses commonly enclose sheets of mafic gneiss (Fig. 3b) that is mainly composed of clinopyroxene (Cpx)+Pl±Amp±Bt±Qtz±Grt. The migmatitic granitic gneisses contain lenticular inclusions of diopside marble, meta-basalt, meta-mudstone, marble and websterite. Figure 2a shows five websterite lenses, the largest of which, labelled No.1, is c. 300-m long and up to 80-m wide. Its western boundary is a thrust that strikes 020° and dips 75° W; the hanging wall is either diopside marble (Fig. 3c) or granitic gneiss. To the west of the thrust a meta-diabase dyke (HS9) transects the foliation of the granitic gneiss (Fig. 3a), and to its east the thrust has transported the gneiss eastwards over the websterite.

The No.1 lens is massive, but internally layered with orthopyroxene (Opx)- and Cpx-rich layers that strike c. 080° and dip c. 25°N, that is, at a high angle to the dominant foliation of the gneiss and marble in the hanging wall and footwall (Fig. 2a,b). Lens No. 1 is heterogeneous, because the modal per cent of Cpx, Opx and other trace minerals changes significantly. The core of the lens is composed of olivine (Ol) websterite (for example, S06-10) in which the dominant Cpx and Opx are accompanied by minor Ol and spinel (Fig. 3d). The eastern side of the lens consists of Grt-websterite (HS12) in which Grt aggregates are up to 5 cm across (Fig. 3e), and Cpx is in contact with Grt or Opx (Fig. 3f), but Ol never coexists with Grt. The lens has been retrogressed under hydrous, low-pressure conditions, as indicated by: (a) partial replacement of Cpx by Amp; (b) symplectites of Opx+Pl+magnetite replacing Grt (Fig. 3g); (c) Cpx contains exsolution lamellae of Opx and has rims of Opx (Fig. 3g). The cores of some Grts contain inclusions of subhedral Amp and Cpx (Fig. 3h), and Opx occurs as inclusions in the cores of some Cpx. Such textural relationships between Grt, Opx, Cpx, Amp and Pl indicate that the Grt-websterite (HS12, Fig. 3f–h) underwent at least three stages of crystallization/metamorphism: the first stage is defined by the inclusions of Amp and Cpx in Grt, the second stage by an anhydrous assemblage of Cpx, Opx and Grt, and the third stage by the symplectitic (Opx+Pl) reaction product between Grt and Cpx.

On the SE margin of No. 1 lens, the Grt-websterite is intruded by a 3 × 5 m meta-gabbro (Fig. 3i), mainly composed of granoblastic Cpx+Pl+Grt+Amp (HS11) (Fig. 3j). Also, many small lenticular meta-gabbro lenses in the granitic gneiss are in thrust contact. These gabbros were also metamorphosed, but differently from the meta-gabbros in the websterite, because they are characterized by Pl+Cpx+Amp+Bt without Grt (S07-3). Figure 2a also shows four small websterites lenses (No. 2–5) that have been imbricated with the granitic gneisses; we observed no Grt in these lenses. Lens No. 4 contains a small (2 × 3 m) meta-gabbro intrusion. Internal mineral layering in lenses No. 3 and 4 strikes parallel to the foliation of the bordering gneiss and dips 50° and 42–65 °N, respectively (Fig. 2c).

**Figure 1:**
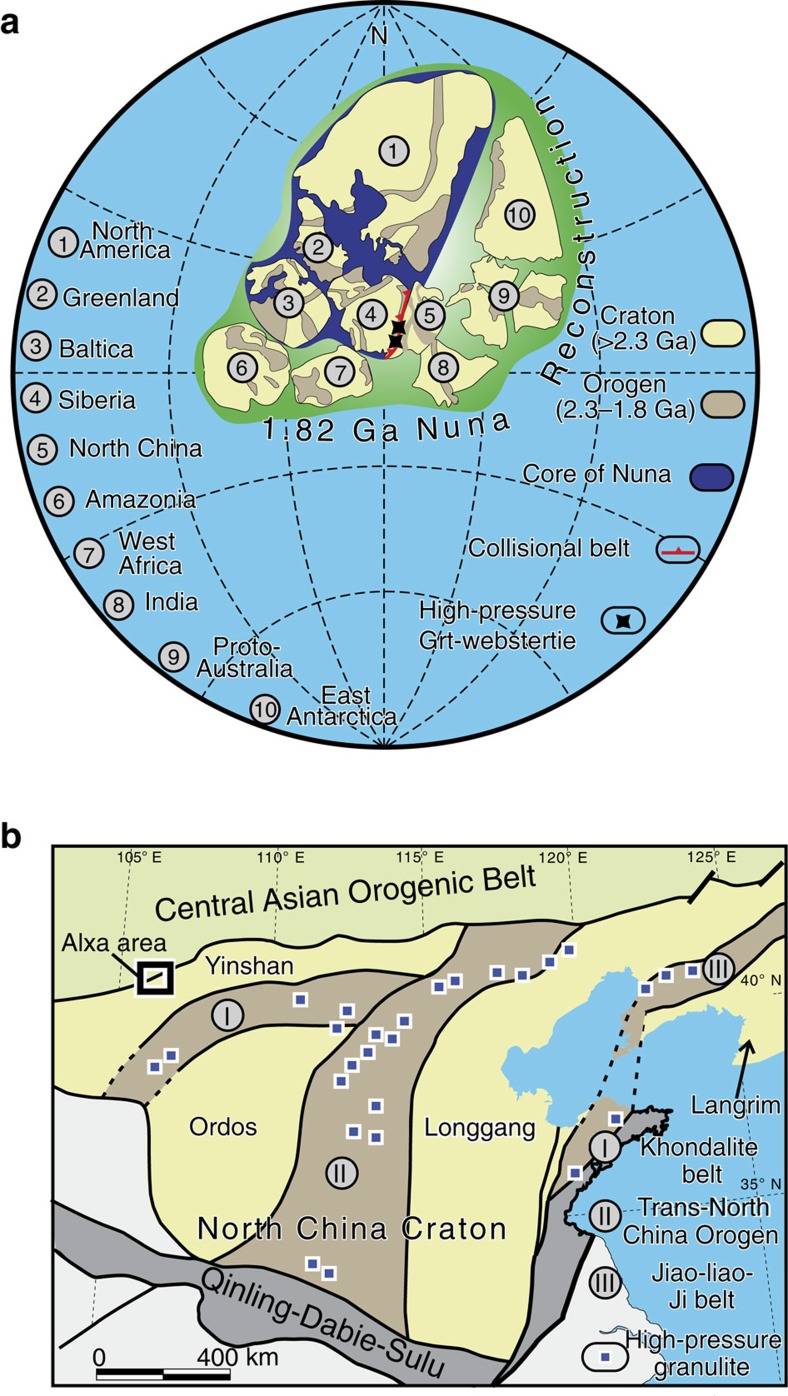
Reconstructed 1.8 Ga Nuna and sketch map of the North China Craton. (**a**) The North China Craton (NCC) and Siberia are in the core of Nuna^11^,^12^. The active margin of SW Siberia and North NCC are juxtaposed, and the Trans-North China orogen continues to the Central Indian Tectonic Zone^10^. (**b**) Yinshan, Ordos, Longgang and Langrim are four Archean blocks that mutually collided to give rise to three Paleoproterozoic orogenic belts within the NCC^19^,^20^. The high-pressure granulite is from Zhao *et al*.^20^ and Zhai *et al*.^21^ The Alxa area (marked in Fig. 2) is on the northernmost margin of the craton.

**Figure 2:**
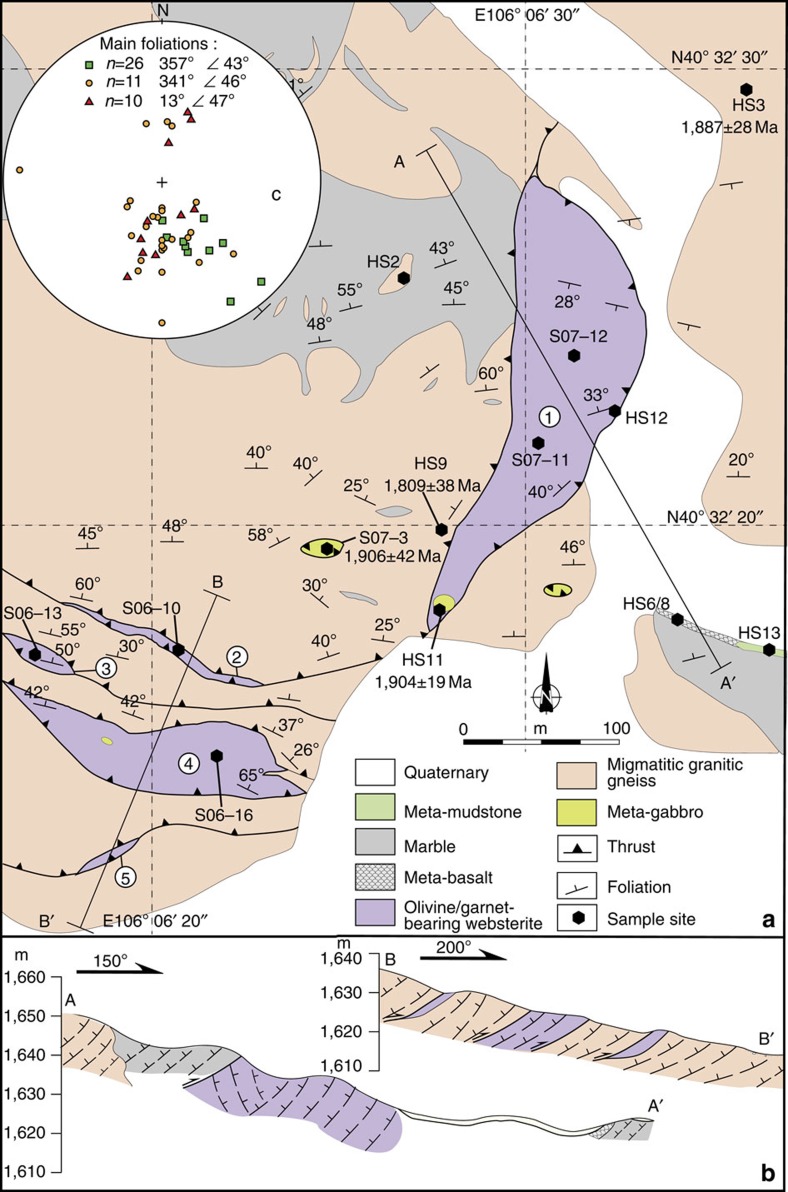
Geological map of the websterite in the Alxa area. (**a**) No. 1 websterite lens contains high-pressure garnet-websterite. (**b**) The cross-sections are indicated on the map and show thrust-imbrication. Sample numbers and ages are marked. (**c**) Stereographic projection with equal-angle plots illustrates East-West-trending and N-dipping foliations. Square=marble, circle=gneiss, triangle=websterite; *n* is number of plotted data.

**Figure 3:**
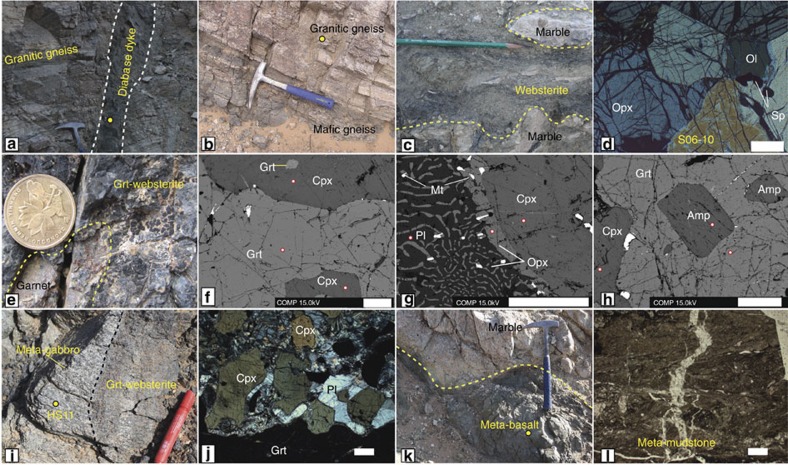
Photographs of typical rocks and minerals with their relationships. (**a**) Meta-diabase (HS9) transects the foliation of granitic gneiss. (**b**) Foliated granitic gneiss and mafic gneiss. (**c**) Fault contact between websterite and marble breccia. (**d**) Ol-websterite with equigranular texture (S06-10). (**e**) Grt-websterite. (**f**) Peak metamorphic stage mineral assemblages with contemporary Grt and Cpx (HS12). (**g**) Retrograde assemblages with symplectite between Cpx and Grt, is composed of Pl, Opx and Mt. A thin Opx rim is along the margin of the Cpx (HS12). (**h**) Grt-websterite with Amp in the core of the Grt defines the pre-peak stage of metamorphism (HS12). (**i**) Meta-gabbro (HS11) intruding websterite. (**j**) Granoblastic Grt, Cpx and Pl in meta-gabbro (HS11). (**k**) Marble on top of meta-basalt (HS6). (**l**) Meta-mudstone with calcite veins. Yellow dots in **a**,**b**,**i** and **k** are sample sites selected for age or geochemical analyses. White dots are analytical spots by EPMA. White bar in **d** is 100 μm, and in **f**–**h**,**j**,**i** it is 500 μm. Amp, amphibole; Cpx, clinopyroxene; Grt, garnet; Mt, magnetite; Ol, olivine; Opx, orthopyroxene; Pl, plagioclase; Sp, spinel.

On the eastern side of Fig. 2a an ESE-striking narrow band of meta-basalt (HS6) is bordered by marble, shown specifically in Fig. 3k. The meta-basalts have a greenschist facies mineralogy characterized by chlorite+epidote, and were intruded by extensive calcite veins. About 30 m along strike to the east of the meta-basalt is a narrow layer of meta-mudstone (Fig. 1), which has a low-grade metamorphic assemblage of aligned muscovite and Qtz. The metamorphic grade of the inclusions of meta-basalt, meta-mudstone and marble is lower than that of the gneisses and Grt-websterite, and yet all these rocks are mutually imbricated by thrusts.

### Whole-rock geochemistry

**Figure 4:**
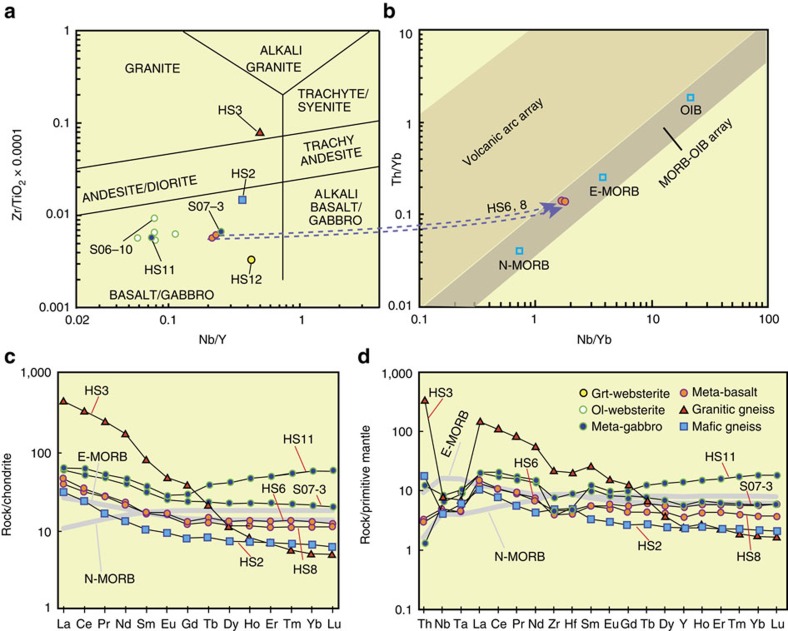
Geochemical plots of different types of rocks in the Alxa complex. (**a**) Rock classification diagram^53^. (**b**) Geochemical discrimination diagram. (**c**) Chondrite-normalized REE diagram and chondrite concentration from Boynton^28^. (**d**) Primitive mantle-normalized multiple trace element diagram and primitive mantle, N-MORB and E-MORB values in **c**,**d** from^29^. Note, only fluid immobile elements have been plotted in **d** because they should not have been affected by metamorphic fluid alteration and can be used to understand the origin of their respective protoliths.

All results are listed in Supplementary Data Set 1. A representative granitic gneiss (HS3) contains 75.23 wt.% SiO_2_, 0.45 wt.% TiO_2_ and 247 p.p.m. Zr. It is enriched in rare earth elements (REE) (after normalization (N) with a chondrite composition^28^); especially the light (L)REE over heavy (H)REE (La/Yb)_N_ ratios are 86, and high field strength element (HFSE)/LREE ratios are low, for example, Nb/La 0.05. The mafic gneiss (HS2) has 50.49 wt.% SiO_2_, 0.56 wt.% TiO_2_ and 55 p.p.m. Zr, and is enriched in LREE (La/Yb)_N_=1.7, and has low HFSE/LREE ratios (for example, Nb/La 0.42). The granitic and mafic gneisses plot in the ‘Granite' and ‘Basalt' fields, respectively (Fig. 4a). The meta-basalts (HS6, 8) have SiO_2_ (47.38–55.88 wt.%), TiO_2_ (1.08–1.13 wt.%) and Zr (56–100 p.p.m.). In Fig. 4b, the basalts plot on the mid-ocean ridge basalt (MORB)-ocean island basalt (OIB) trend, and are close to enriched (E)-MORB. The meta-basalts have a slight enrichment in LREE (La/Yb)_N_=3.17–3.57, similar to that of E-MORB^29^ (Fig. 4c), whereas they are depleted in HFSE after normalization with a primitive mantle composition^29^ (Fig. 4d).

The websterites are characterized by very low SiO_2_ (41.37–44.23) wt.%, and the TiO_2_, Al_2_O_3_, MgO and FeO contents change significantly between Grt-websterite (HS12) and Ol-websterites (for example, S06-10). The Grt-websterite has low MgO (8.89 wt.%), and high TiO_2_ (2.68 wt.%), Al_2_O_3_ (12.49%), Fe_2_O_3_T (22.59 wt.%) and Zr (91 p.p.m.) in contrast to the Ol-websterites, which have high MgO (23.17–26.35 wt.%), and low TiO_2_ (0.35–0.53 wt.%), Al_2_O_3_ (5.89–6.89%), Fe_2_O_3_T (11.47–12.10%) and Zr (22–34 p.p.m.). On Fig. 4a, all websterites plot in the ‘Basalt' field.

The meta-gabbros (HS11, S07-3) have 44.34–46.96 wt.% SiO_2_, TiO_2_ (0.82–1.56 wt.%) and Zr (46–89 p.p.m.). They have a slight enrichment in LREE (La/Yb)_N_=1.42–3.32, and low HFSE/LREE ratios (for example, Nb/La 0.34–0.44). The Grt-rich meta-gabbro (HS11) has an elevated HREE (Fig. 4c).

### P–T conditions of the Grt-websterite

Grt-websterite contains the best evidence of HP metamorphism in the area; we chose the least retrogressed sample (HS12) to constrain the pressure–temperature (P–T) path (Table 1), and mineral compositions used for the P–T calculations for each stage are listed in Supplementary Data Set 2.

**Figure 5:**
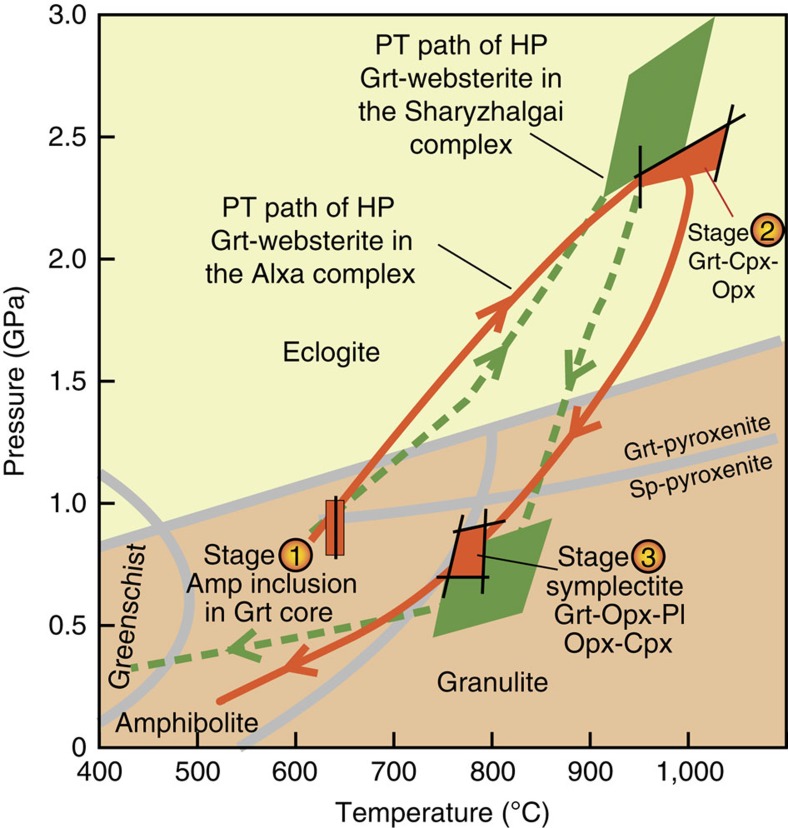
P–T diagram of the HP Grt-websterite in the Alxa area. Grt–Sp transition curve for websterite (pyroxenite) is from Herzberg^30^, and the metamorphic facies is from Bucher and Grapes^36^. Results of P–T calculations for studied samples are shown as assemblages of two to four reaction curves for geothermobarometry, listed in text and Table 1. The line with arrows is a proposed clockwise P–T trajectory. The P–T trajectory of the Grt-websterite of the Sharyzhalgai complex is from Ota *et al*.^59^.

The mineral assemblages/reactions in the first stage do not constrain the pressure; the Grt in the websterite is stable only at a higher pressure of >0.9 GPa^30^. Therefore, the minimum pressure for the first stage should be ∼0.9 GPa, and the temperature constrained by the Amp-Grt reaction^31^ is 639 °C (Fig. 5, Table 1); the second stage represents the peak P–T metamorphism indicated by the anhydrous minerals. As mentioned above, the rocks have been variably retrogressed, so only the cores of Grt, Opx and Cpx (Fig. 3b) were used to obtain the P–T conditions, as close to the maximum as possible; however, even these values should be regarded as minimum estimates. The relevant mineral compositions yield the highest P–T conditions of 2.37 GPa^32^ at 1,000 °C^33^. Grt–Cpx and Grt–Opx geothermometries give temperatures of 934 °C^34^ and 1,045 °C^33^, respectively (Fig. 5). The third stage is constrained by minerals associated with the symplectite (Fig. 3c). Geothermobarometry using the reactions of Grt–Cpx–Opx–Pl^35^ and Grt–Opx^32^ yields pressures of 0.65 and 0.84 GPa, respectively, and Grt–Opx^33^ constrains the temperature as 756–796 °C (Fig. 5). These three stages of the metamorphism of the Grt-websterite with their estimated P–T conditions define a clockwise P–T path (Fig. 5), which outlines a significant P–T increase up to the eclogite-facies^36^, then exhumation to the granulite facies and finally to the surface.

### Zircon U-Pb geochronology

**Figure 6:**
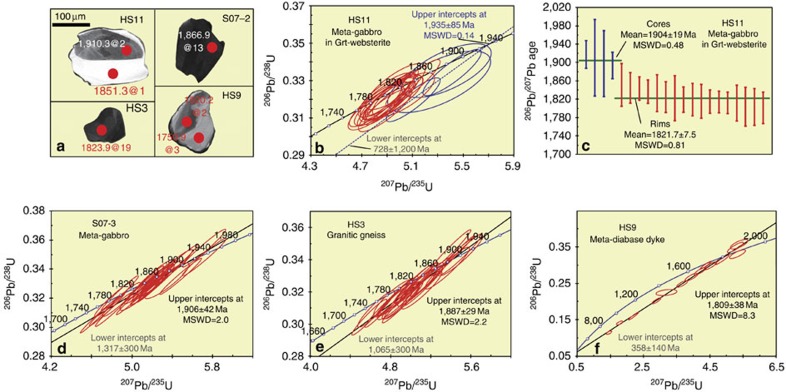
CL images of zircons and U-Pb concondia diagrams. (**a**) The red circles are analytical spots; age@ analytical order are from the Supplementary Data Set 3; the bar, 100 μm. (**b**) U-Pb concordia diagram of meta-gabbros (HS11). (**c**) Weighted average mean age diagram of meta-gabbros (HS11). (**d**–**f**) U-Pb concordia diagrams of meta-gabbro (S07-3), granitic gneisses (HS3) and meta-diabase (HS9), respectively.

U-Pb results of four samples are listed in Supplementary Data Set 3. The meta-gabbro (HS11), intruding Grt-websterite, was chosen for constraining the ages of both rocks. Most zircons have core-rim textures and are round, and their cores are anhedral and show patch textures in CL image (Fig. 6a); this morphology suggests that the overgrowth rims are metamorphic^37^. Four cores and 17 rims were analysed, and all results plot close to the concordia curve of ^206^Pb/^238^U versus ^207^Pb/^235^U (Fig. 6b). Four cores yield a discordia line that intersects with the concordia curve at an upper intercept of 1,935±85 Ma with a mean square weighted deviation (MSWD) of 0.14 and a lower intercept of 728±1,200 Ma (Fig. 6b), whereas ^206^Pb/^207^Pb ages yield a weighted mean age of 1,904±19 Ma with a MSWD of 0.48 with Isoplot^38^ (Fig. 6c). Results from the rims did not yield a concordia age nor a valid discordia line, but the ^206^Pb/^207^Pb ages give a weighted mean age of 1,821.7±7.5 Ma with a MSWD of 0.81 (Fig. 6c).

The meta-gabbro lens (S07-3) that is in thrust contact with granitic gneiss contains zircons that are all anhedral, and a few show a zircon zonation typical of mafic magma (Fig. 6a). Most zircons are dark and do not have clear core-rim textures (Supplementary Fig. 2). About 25 analyses on 25 grains follow a discordia line that intersects with the concordia curve at an upper intercept of 1,906±42 Ma with a MSWD of 2.0 and a lower intercept of 1,317±300 Ma (Fig. 6d).

Zircons from the granitic gneiss (HS3) are dark and anhedral; some show magmatic zonation (Fig. 6a), and a few have core-rim textures, but the rims were too narrow for U-Pb analyses. About 22 results plot along a discordia line that intersects with the concordia curve at an upper intercept of 1,887±29 Ma with a MSWD of 2.2 and a lower intercept 1,065±300 Ma (Fig. 6e).

The meta-diabase dyke (HS9) that transects the foliation of the granitic gneiss (Fig. 3a) was analysed to constrain the times of diabase crystallization and granite deformation; but not many zircons were separable from the sample. They are mostly round and have core-rim textures (Fig. 6a). The sample was also metamorphosed, because its core age is even younger than its rim age (Fig. 6a). All results define a steep discordia line that plots far off the concordia curve, which intersects with the concordia curve at an upper intercept of 1,809±38 Ma Ma with a MSWD of 8.3 and a lower intercept of 358±140 Ma (Fig. 6e).

## Discussion

All tectonothermal events took place in the Paleoproterozoic. All rocks underwent different degrees of metamorphism, as illustrated by the zircon morphology and age results (Fig. 6b–f). Many studies have demonstrated that the upper intercept of discordant ages indicates the crystallization age of zircons^26^. On the other hand, discordant lower intercept ages are problematic, and most have no geological meaning^39^; therefore we ignored the lower intercept ages in this study. For the meta-gabbro (HS11) that intruded the Grt-websterite, the upper intercept age of 1,935±85 Ma of the zircon cores is consistent with the weighted mean age of 1,904±19 Ma within error. Because the weighted mean age has a smaller error and a MSWD closer to 1, we interpret the ∼1,904 Ma age as the time of crystallization of the gabbro, and therefore the websterite must be older than ∼1,904 Ma according to the geological relationships. Also, the high-quality weighted mean age of the overgrowth of the zircon rims in HS11 is ∼1,822 Ma. Thus, we consider it most likely that the gabbro intruded the websterite before the peak metamorphism of the websterite, which has a zircon core age of 1,904 Ma, so the HP metamorphism must have occurred between 1,904 and 1,822 Ma. During exhumation from the mantle to the deep crust both rocks underwent retrogression at 1,822 Ma, when the zircon overgrowth rims formed. Accordingly, the time between subduction to the mantle and the time of exhumation and emplacement in the deep crust was ∼80 Ma. This time-range between subduction and exhumation is comparable to that in many orogenic belts such as the Chinese Tianshan^40^. Generally, the exhumation of HP rocks is fast, because slow exhumation tends to erase the HP fingerprint^41^.

The meta-gabbro (S07-3) has an upper intercept of 1,906±42 Ma, which we interpret as the time of crystallization; significantly this is close to the crystallization age of the sample HS11, which in turn suggests that all the gabbros were contemporaneous. The granitic gneiss that encloses the gabbroic and websterite lenses has an upper intercept age of 1,887±28 Ma, which we consider to be the time of crystallization of the granite. The age of 1,887 Ma is younger than the ages of the enclosed gabbroic lens (S07-3 ∼1906 Ma) and websterite lens (>1,904 Ma); this provides chronological confirmation of the fact that the gabbro and websterite are in tectonic contact with the gneiss (Fig. 2a,b). The meta-diabase (HS9) has an upper intercept age at 1,809±38 Ma, which we interpret as the time of crystallization. This dyke clearly transects the foliation of the granitic gneiss (Fig. 3a), so it must post-date the ductile deformation of the gneiss. Also, the 1,809 Ma dyke underwent mild metamorphism, and we note that a 1.79 Ga metamorphic event has been recorded farther east in the Alxa area^42^. Therefore, we consider this was most likely responsible for the metamorphism of the dyke.

**Figure 7:**
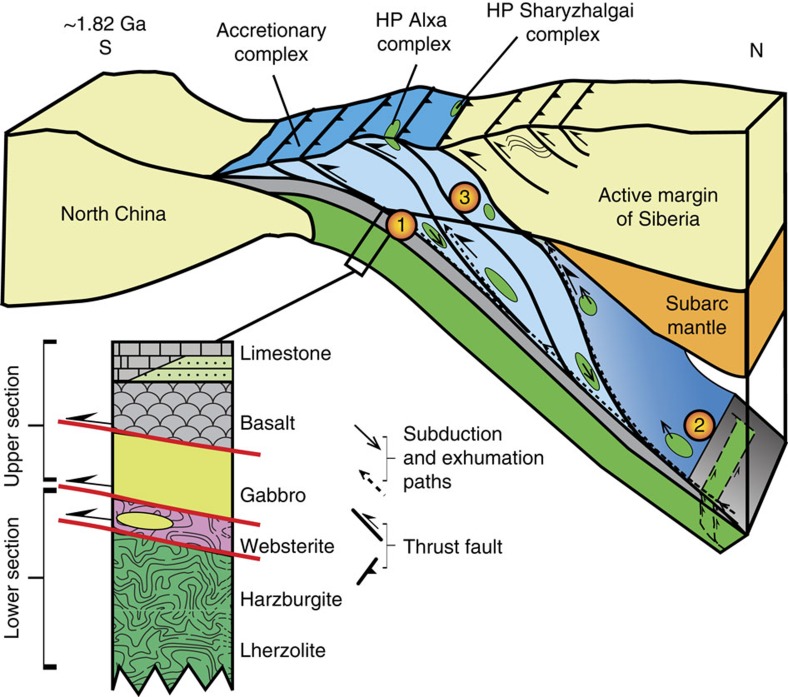
The tectonic setting of the Alxa HP Grt-websterite. The cartoon illustrates the situation when the northern margin of the NCC collided with the southern Siberian craton at∼1.82 Ga. Websterite in oceanic crust (ophiolite) with sedimentary and other crustal rocks (marked by 1) is subducted to depth in the mantle giving rise to HP metamorphism of the Grt-websterite at a pressure of ∼2.4 GPa (marked by 2). The HP rocks were exhumed to the lower crust where granulite facies conditions record ∼0.9 GPa pressure at 1.82 Ga (marked by 3). The possible positions of the HP Alxa and Sharyzhalgai complexes are suggested. The subduction–exhumation paths from 1 to 2 to 3, correlate with those in the PT diagram in Fig. 5. The reconstructed pseudostratigraphy of the ultramafic rocks, gabbro, basalt, mudstone and limestone is comparable to that of an ophiolite. The harzburgite and lherzolite are from location C in Supplementary Fig. 1.

As reported above, the websterite and meta-gabbros were imbricated and repeated by thrusting with granitic gneiss, meta-basalt, meta-mudstone and marble. Similar lithologies and structural relations are characteristics of many ophiolites^43^, which include gabbro, basalt and pelagic sediments that were typically thrust-imbricated^44^ during tectonic accretion^43^. Such ophiolites may have formed in a MOR^45^, but geochemical and structural studies have suggested that most ophiolites formed in a supra-subduction zone (SSZ) setting^46^,^47^. Although, the mudstone, marble and underlying basalt are in mutual thrust contact in the field (Fig. 3f), they are comparable to ocean plate stratigraphy in which similar lithologies are usually separated by thrusts^48^. Although websterite and gabbro are the only ultramafic-mafic rocks in the study area, many lenses of harzburgite, lherzolite, websterite, gabbro and basalt crop out at location C (on Supplementary Fig. 1), only 25 km to the southwest along strike of this study, where lherzolite and websterite are Grt bearing. All these ultramafic-mafic lenses are widespread and concentrated along a NE-trending belt throughout the Alxa area, and they are commonly accompanied by marbles^27^. Taken together, the lithologies and associations point towards the reconstructed stratigraphy of an ophiolite^44^ (Fig. 7).

Geochemically, the meta-basalts (HS6, 8) and meta-gabbros (HS11, S07-3) are characterized by a depletion of HFSE (Fig. 4d), which is indicative of a subduction-related setting in contrast to modern N-MORB oceanic basalts. These arc-type websterite-gabbro-basalt rocks at Alxa are suggestive of an incipient SSZ ophiolite. The Amp inclusions in Grt (Fig. 3d) are characterized by high TiO_2_ (2.79 wt.%) and a low temperature of 639 °C, which is similar to that of magmatic Amp in mafic magmatic rocks^49^, in contrast to metamorphic Amps that have low TiO_2_. These relic magmatic high-Ti Amps provide additional support for a SSZ origin, and also Amp is a diagnostic indicator of hydrous island arcs or active continental margin arcs^50^, as further constrained by many experimental studies^51^. The contrasting metamorphic grades of the websterites and meta-basalts suggest the likely presence of an intervening thrust or detachment (Fig. 7). In a subduction zone, most rocks from the lower oceanic crust are transported to the mantle with the result that most of the upper and a little of the lower oceanic crust are peeled off and stacked in the toe of an arc in an accretionary wedge^52^. Like the meta-gabbros, the granitic (HS3) and mafic (HS2) gneisses are characterized by depletion of HFSE, which is a signature of subduction. A short time-gap between magmatic and metamorphic events is a common feature of many arcs^53^. During subduction some rocks may be transported to mantle depth to undergo HP metamorphism, in which case Grt-websterites may be expected after exhumation. Sedimentary rocks and granites (*ρ*∼3 g cm^−3^) are less dense than ultramafic rocks (*ρ*∼3.3 g cm^−3^), and thus are relatively buoyant and would assist exhumation. An accretionary complex develops in a trench as a result of oceanic plate subduction, and is preserved between a downgoing (oceanic or continental) plate and a hanging wall plate, both of which may contain an arc, and the exhumation of HP rocks takes place within an extruded wedge^54^,^55^. In this study, the HP Alxa rocks are situated next to low metamorphic grade rocks, a common relationship in both modern and ancient orogenic belts^56^,^57^. The exhumation of the Alxa HP rocks may have been facilitated by buoyant granitic rocks into an accretionary complex.

Today there is no contemporary geological terrane to the north of the Alxa complex (at E 106°, N 40°), instead there is the Paleozoic Central Asian Orogenic Belt (Fig. 1b). Therefore, predictably the corresponding overriding terrane has been removed. Within an accretionary complex the predominant structures characteristically dip towards to the overriding plate, and this is a diagnostic indicator of the sense of subduction polarity. As shown in Fig. 2c, most foliations dip to the N, suggesting that an overriding arc was located to the north of the Yinshan block in the Paleoproterozoic. The most likely candidate for this ‘lost' terrane is the Siberian craton, the southwestern margin of which contains Paleoproterozoic HP ultramafic-mafic complexes such as the Sharyzhalgai at c. E 104°, N 56° (ref. 58). Indeed, the lithologies, structural relations, metamorphic grades, PT trajectories and isotopic ages of the Sharyzhalgai and Alxa complexes are so remarkably similar (Fig. 5 and Supplementary Data Set 2), including the presence of identical Grt-websterites^59^, that it is hard to avoid the conclusion that these two complexes did not undergo comparable subduction–exhumation processes in the same Paleoproterozoic orogenic belt. There is documentary evidence that the southwestern margin of the Siberian craton was an active subduction zone in the Paleoproterozoic, when a craton from the south was accreted to it^59^,^60^. However, in the latest reconstruction of the evolution of Nuna^11^, the southwestern margin of Siberia did not collide throughout its history with any other craton, and did not terminate with any collided craton to the south. This tectonic scenario is difficult to accept and rationalize, because the intervening oceanic plate between any two cratons must be removed by subduction before the orogenesis is terminated by arc–continent or continent–continent collision. We propose an alternative, more viable, model that the northern margin of the NCC was finally attached to the southwestern margin of the Siberian craton at ∼1.82 Ga. This model is supported by: (1) Paleomagnetic data^15^, which indicate that the northern margin of the NCC had the same polar wonder path as the southern margin of the Siberian craton from ∼1.8 to 1.3 Ga, when the two craton margins were in mutual contact. (2) The occurrence of ∼1.7 Ga anorogenic rapakivi granites (with gabbros and anorthosites) 61,62 and mafic dykes^16^ in both craton margins demonstrates that they both underwent a similar post-orogenic event.

The connections, positions and correlations between the NCC and other continents in the context of the Nuna reconstruction are highly debated^10^,^13^,^14^,^15^,^16^, in particular with regard to the relative positions of India or Baltica^10^,^13^. We can find no support for the speculation that the northern margin of the NCC was in a within-plate setting in the Paleoproterozoic^10^. Alternatively we argue that, although the NCC and Siberian craton have somewhat different basements, they were juxtaposed by closure of an intervening ocean; the dissimilarity of two basements does not preclude their connection. A recent global paleomagnetic synthesis^11^ has provided a new configuration of continental blocks within Nuna, in which the NCC was separated from Siberia by India; also in the global model of Zhao *et al*.^10^, NCC-India was separated from Siberia by other continents. Our new Paleoproterozoic reconstruction more satisfactorily explains: (1) the active HP margin of the northern NCC against the coeval HP active margin of the southwestern margin of Siberia, and (2) the position of the NCC against India. This configuration allows connection and continuity between the coeval Trans-North China orogen and the Central Indian Tectonic zone^10^ (Fig. 1a), and significantly it is supported by the occurrence of Paleoproterozoic to Mesoproterozoic dyke swarms that stitched the southern NCC and southern India^14^. Our reconfiguration mainly focuses on the framework of Paleoproterozoic Nuna, and later positions of continental blocks were predictably different from the 1,740 Ma configuration^11^ because of movements of plates with time.

## Methods

### Whole-rock analyses

Rock chips were ground in an agate mill and prepared for whole-rock analysis. Major element oxides were analysed on fused glass disks with a Phillips PW 1500 X-ray fluorescence spectrometer. The precision and accuracy of the major element data were determined with Chinese whole-rock basalt standards: GSR-3≤5% and *ca.* 5% (2σ), respectively. FeO concentrations were determined by titration and the loss on ignition gravimetrically. The chemical analyses were carried out at the Institute of Geology and Geophysics, Chinese Academy of Sciences (IGGCAS).

Trace elements were determined by inductively coupled plasma mass spectrometry (ICP-MS; VG-PQII) at IGGCAS. Sample powders were decomposed in a mixture of distilled HF-HNO_3_ in Savillex Teflon beakers for 6 days at 120 °C. The sample solution was dried and the residue dissolved in 50 ml 1% HNO_3_ for ICP-MS analysis. Indium was used as an internal standard for correction of matrix effects and instrumental drift. The procedural blank contribution for the trace elements is ≤446 pg, which is not significant, considering the high concentrations of incompatible elements in the granitoids. The precision and accuracy of the data are better than 5% as determined on GSR-3.

### Major oxides of minerals

Rock chips were mounted on glass, and polished to 30-μm thickness for thin sections for mineral composition analyses. Garnet, pyroxene, Amp and Pl compositions were determined at IGGCAS using a JEOL JXA-8100 electron microprobe; the operating conditions were in wavelength-dispersive mode. Analyses were made with a 1–2 μm beam spot size, a 15 kv accelerating voltage, a counting time of 20 s and a 20 nA beam current per element. Microprobe analytical standards were jadeite for Na, Al, and Si; Ol for Mg; synthetic chromite for Cr; chrome diopside for Ca; orthoclase for K; rutile for Ti; rhodonite for Mn and Grt for Fe. A programme based on the ZAF procedure was used for data correction. The precision and accuracy of the data are better than 5%.

### Ion microprobe for zircon ages

Measurements of U, Th and Pb isotopes of zircon were conducted using a Cameca-IMS 1280 large-radius SIMS at IGGCAS. Zircon U–Th–Pb ratios and absolute abundances were determined relative to the standard zircon 91500 (ref. 63), analyses of which were interspersed with those of unknown grains, using operating and data processing procedures described in detail in the study by Li *et al*.^64^. A long-term uncertainty of 1.5% (1 relative standard deviation (RSD)) for ^206^Pb/^238^U measurements of the standard zircons was propagated according to the unknowns in the study by Li *et al*.^65^, despite the fact that the measured ^206^Pb/^238^U error in a specific session was generally about 1% (1 RSD) or less. Measured compositions were corrected for common Pb using non-radiogenic ^204^Pb. The corrections were sufficiently small to be insensitive to the choice of common Pb composition, and an average of present-day crustal composition^66^ was used for the common Pb assuming that it was largely surface contamination introduced during sample preparation. Uncertainties of individual analyses in the data tables are reported at 1σ level, and the age calculations by Isoplot are at 2σ level. Each analytical spot was about 30 μm.

## 

## Additional information

**How to cite this article:** Wan, B. *et al*. Paleoproterozoic high-pressure metamorphism in the northern North China Craton and implications for the Nuna supercontinent. *Nat. Commun.* 6:8344 doi: 10.1038/ncomms9344 (2015).

## Supplementary Material

Supplementary Information Supplementary Figures 1-2

Supplementary Data 1 Major- and trace-element concentrations of representative rocks from the Alxa complex

Supplementary Data 2 Representive mineral compositions of the garnet-websterite (HS12) from the Alxa complex

Supplementary Data 3 SIMS zircon U-Pb data of representative rocks from the Alxa complex

## Figures and Tables

**Table 1:** P–T estimates of the garnet-websterites in the Alxa area.

	**Metamorphic stages**		
	**Stage 1**	**Stage 2**	**Stage 3**
**Geothermo barometer**	**Amp inclusion in Grt core**	**Grt–Cpx**–**Opx**	**Symplectite (Grt–Cpx–Pl+Opx)**
Grt–Amp(*T*)^31^	639 °C	—	—
Grt–Opx(*P*)^32^	2.37 GPa		—
Grt–Opx(*T*)^33^	—	1,048 °C	
Grt–Opx(*T*)^34^	—	934 °C	
Grt–Cpx–Opx–Pl(*P*)^35^	—	—	0.65 GPa
Grt–Opx(*P*)^32^	—	—	0.84 GPa
Grt–Opx(*T*)^33^	—	—	756 °C

Amp, amphibole; Cpx, clinopyroxene; Grt, garnet; Opx, orthopyroxene; *P*, pressure; Pl, plagioclase; *T*, temperature.

Mineral compositions, for calculations, are listed in Supplementary Data Set 1. The uncertainties estimated for Stage 2 (peak P–T condition) are about 0.3 GPa and 80° C (refs 32, 34), respectively, and for other stages are better than 0.1 Gpa and 50 °C (refs 31, 35).
