# Supplementary material for: Paleoproterozoic high-pressure metamorphism in the northern North China Craton and implications for the Nuna supercontinent
Source: Nat Commun. 2015 Sep 21;6:8344. doi: 10.1038/ncomms9344 (PMC4595726; doi:10.1038/ncomms9344)
Supplement: Supplementary Information — Supplementary Figures 1-2 [file ncomms9344-s1.pdf]

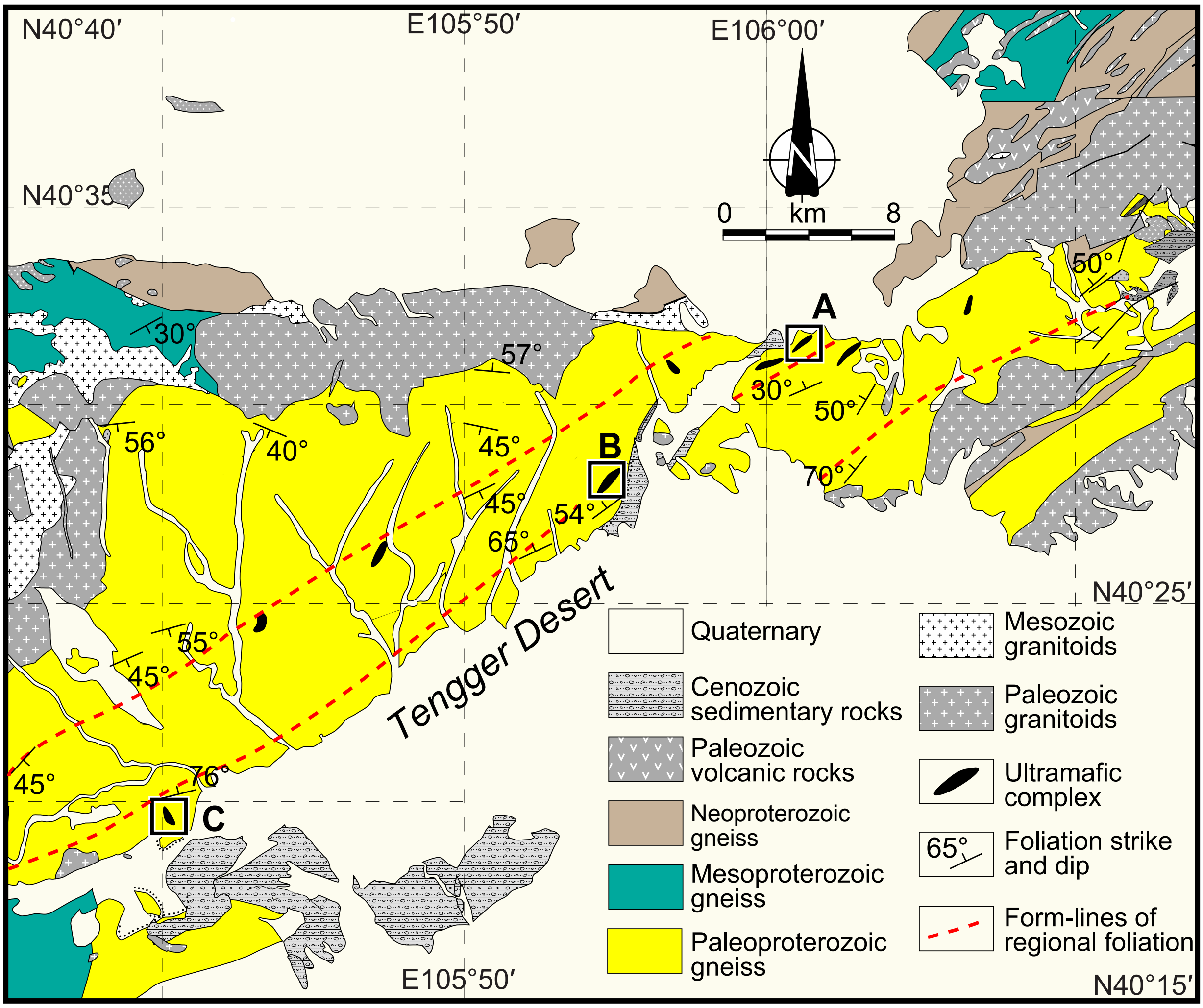

Supplementary Figure 1. Geological map of the Alxa area. A marks our study area, and B and C mark other ultramafic-mafic complexes along strike, referred to in the text.

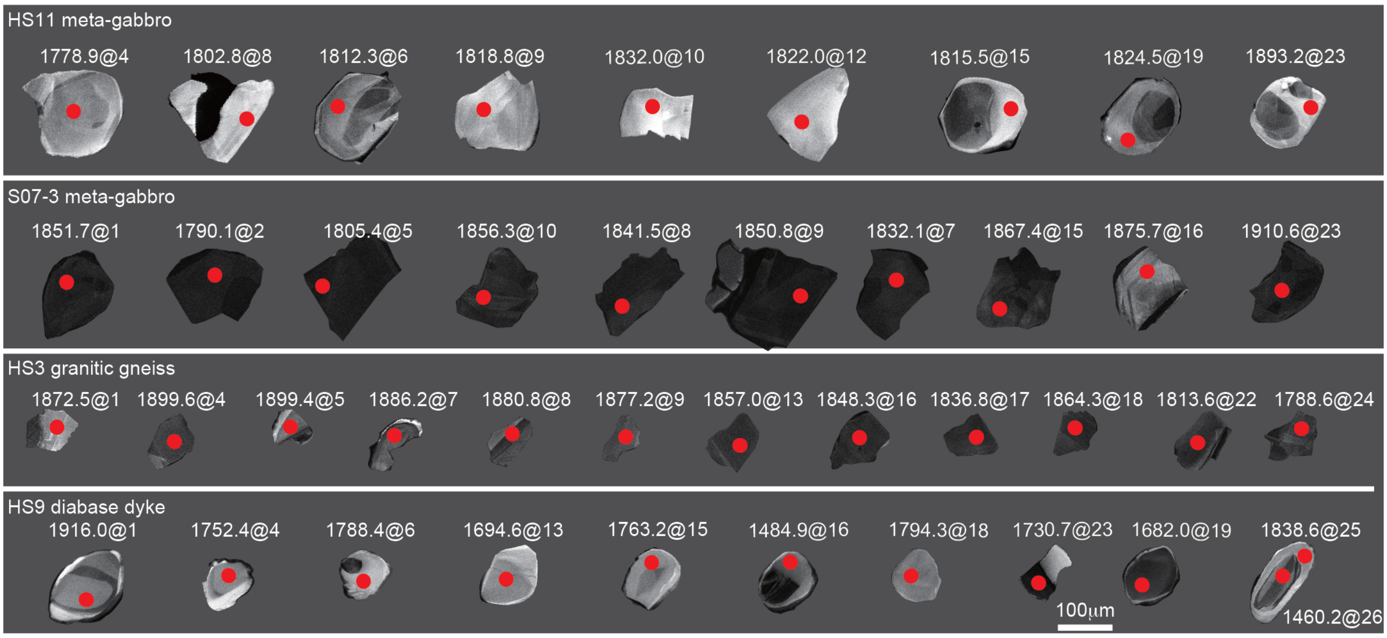

Supplementary Figure 2. CL images of typical zircons. The red circles are analytical spots, age@ analytical order are from the Supplementary Data Set 3.
